# School and community driven dengue vector control and monitoring in Myanmar: Study protocol for a cluster randomized controlled trial

**DOI:** 10.12688/wellcomeopenres.18027.1

**Published:** 2022-08-10

**Authors:** Hans J. Overgaard, Nay Yi Yi Linn, Aye Mon Mon Kyaw, Leo Braack, Myo Win Tin, Sheri Bastien, Fiona Vande Velde, Pierre Echaubard, Win Zaw, Mavuto Mukaka, Richard Maude

**Affiliations:** 1 Faculty of Science and Technology, Norwegian University of Life Sciences, As, 1432, Norway; 2 Department of Microbiology, Faculty of Medicine, Khon Kaen University, KHON KAEN, 40002, Thailand; 3 Central Vector Borne Disease Control Unit, Ministry of Health and Sports, Nay Pyi Taw, Myanmar; 4 Yangon Regional Health Department, Ministry of Health and Sports, Yangon, Myanmar; 5 Malaria Consortium, Bangkok 10400, Thailand; 6 Institute for Sustainable Malaria Control, University of Pretoria, Pretoria 0028, South Africa; 7 Malaria Consortium, Yangon 11041, Myanmar; 8 Faculty of Landscape and Society, Norwegian University of Life Sciences, 1432 Ås, Norway; 9 Department of Community Health Sciences, Cumming School of Medicine, University of Calgary, Calgary, Alberta, T2N 1N4, Canada; 10 School of Oriental and African Studies (SOAS), University of London, London, WC1H 0XG, UK; 11 Faculty of Environment and Resource Studies, Mahidol University, Salaya, 73170, Thailand; 12 Mahidol-Oxford Tropical Medicine Research Unit, Faculty of Tropical Medicine, Mahidol University, Bangkok 10400, Thailand; 13 Centre for Tropical Medicine and Global Health, Nuffield Department of Medicine, University of Oxford, Oxford, OX3 7LG, UK; 14 Harvard T.H. Chan School of Public Health, Harvard University, Boston, MA, 02115, USA

**Keywords:** Dengue incidence, school, KAP, community engagement, implementation fidelity, Aedes

## Abstract

**Background.** Dengue is the most common and widespread mosquito-borne arboviral disease globally estimated to cause >390 million infections and >20,000 deaths annually. There are no effective vaccines or preventive drugs. Control of dengue transmission relies primarily on mosquito vector control. Although most vector control methods currently used by national dengue control programs may temporarily reduce mosquito populations, there is little evidence that they affect transmission. There is an urgent need for innovative, participatory, effective, and locally adapted approaches for sustainable vector control and monitoring in which students can be particularly relevant contributors and to demonstrate a clear link between vector reduction and dengue transmission reduction, using tools that are inexpensive and easy to use by local communities in a sustainable manner.

**Methods.** Here we describe a cluster randomized controlled trial to be conducted in 46 school catchment areas in two townships in Yangon, Myanmar. The outcome measures are dengue cases confirmed by rapid diagnostic test in the townships, dengue incidence in schools, entomological indices, knowledge, attitudes and practice, behavior, and engagement.

**Conclusions.** The trial involves middle school students that positions them to become actors in dengue knowledge transfer to their communities and take a leadership role in the delivery of vector control interventions and monitoring methods. Following this rationale, we believe that students can become change agents of decentralized vector surveillance and sustainable disease control in line with recent new paradigms in integrated and participatory vector surveillance and control. This provides an opportunity to operationalize transdisciplinary research towards sustainable health development. Due to the COVID-19 pandemic and political instability in Myanmar the project has been terminated by the donor, but the protocol will be helpful for potential future implementation of the project in Myanmar and/or elsewhere.

Registration: This trial was registered in the ISRCTN Registry on 31 May 2022 ( https://doi.org/10.1186/ISRCTN78254298).

## Introduction

Dengue is the most common mosquito-borne arboviral disease in the world with an estimated >390 million infections and >20,000 deaths annually and about 2.5 billion people living in risk areas ^1^. Dengue is caused by a flavivirus (DENV) consisting of four serotypes which offer no cross-protective immunity, meaning that an individual can contract dengue fever up to four times in a lifetime. Dengue and other arboviruses, such as Zika and chikungunya, are transmitted primarily by the *Aedes aegypti* mosquito vector, which breeds preferentially in artificial water containers near human habitation. *Aedes albopictus* is considered a secondary vector, more abundant in rural areas. Both vectors are day-biting mosquitoes ^2^. A tetravalent dengue vaccine has been developed but offers only incomplete protection and is associated with adverse events in seronegative individuals ^3^. As there are no current therapeutic and preventive drugs available ^4^, vector control remains the primary method to prevent DENV transmission. Vector control with a focus on the most productive, and hence epidemiologically important, mosquito breeding sites is cost-effective, especially in resource-constrained settings and in disease hotspot areas ^5, 6^. This requires a sound knowledge of local vector ecology, household water management practices, and spatial variations in disease patterns.

The incidence of reported dengue doubles approximately every decade ^7^. In the 1960’s, dengue was present in nine countries, but today occurs in at least 128 countries ^8^. The global and local driving forces responsible for this increase are rapid, unplanned, and unregulated urban development, globalization, population growth, climate, inadequate municipal services, insecticide resistance, and lack of sustainable and integrated interventions that account for the complexities of ecological and social systems ^9– 12^. Despite increased research and knowledge about factors affecting transmission of arboviruses, effective and sustainable disease control is still lacking. Disease and vector control have long relied on vertically structured single interventions with limited or nonexistent community involvement without recognizing the importance of complex adaptive social-ecological systems that includes pathogen-vector-host-environment relationships ^9, 13^. There is a compelling need for integrated, innovative, effective, and locally adapted approaches for sustainable vector control. Engaging schools and students and promoting ownership of disease control interventions have high potential for achieving sustainable change to reduce dengue transmission as engaged local actors can sustain interventions after an externally funded project terminates ensuring continuity of project outcomes.

Infectious diseases, such as those caused by arboviruses, are likely to spread easily in schools due to frequent contact of students with infective resident mosquitoes breeding in and near the school ^14, 15^. Students are disproportionately exposed to the day-biting habits of *Aedes* mosquitoes compared to other age groups. A study in Mexico revealed that students, teachers, and other personnel were more likely to be exposed to DENV-infected *Ae. aegypti* females on school premises ^14^. Schools also provide a cost-effective entry point for dengue prevention and control that can enhance community-wide vector control through knowledge transfer from schools to communities ^16^. A combination of vector control tools and pedagogical approaches can improve knowledge uptake as well as knowledge and technology transfer from classrooms to homes ^16^. This is important because dengue predominantly affects children ^2^, and vector control measures such as mosquito source reduction require constant household and community engagement.

Schools have been engaged in national dengue control programs in several countries and school-based vector control trials have been associated with increased dengue knowledge ^17, 18^, improved prevention/control practices in schools ^17, 19^, and contributed to improved school and community-based vector control activities ^18^. A trial in Colombia showed that schools receiving sets of interventions targeting dengue (and diarrhea) risk factors reduced mosquito breeding in the schools and significantly improved knowledge in children ^19, 20^ and their parents [Sarmiento-Senior, 2022 submitted]. In Puerto Rico ^21^ and in Thailand ^22^, primary school programs have succeeded in increasing children’s knowledge of, and participation in, dengue prevention and control. Similarly, a school-based dengue control program in Honduras increased knowledge of both the cause of dengue and the vector life cycle, leading to increased participation in controlling larval breeding sites and the consequent reduction of the number of sites ^17^. An ethnographic study conducted in eastern Cambodia demonstrated partial adherence in routine education activities and the need for approaches to ensure the translation of knowledge into practice ^23^. However, a study among university students in the Philippines showed that students were well aware of the types of mosquito larval habitats but did not participate much in source reduction activities ^24^, i.e., school-based interventions are useful to improve knowledge, but not necessarily practices. This reflects the well-known gap between knowing and acting ^25^.

School-based dengue interventions sometimes engage the wider community as part of the intervention. Outcomes generally relate to improved knowledge, attitudes, and practice (KAP) scores, reduced entomological indices, or community empowerment. Few studies show an effect on epidemiological outcomes ^18^. A World Health Organization (WHO)-funded pilot project in Thailand using a combination of top-down and bottom-up approaches found that serologically-confirmed cases were reduced in control areas ^9^. Similarly, risk of dengue virus infection in children was shown by serological evidence from intervention sites in Mexico and Nicaragua ^26^. Most studies reporting encouraging results showed that interventions were rarely sustainable ^18, 20^. The effectiveness of school-based measures in sustainable reduction of vectors or dengue transmission in the wider community remain to be documented.

Myanmar is a low-income country in Southeast Asia with a population of approximately 52 million, of which 6.1 million are children <five years of age. Dengue is a priority disease in Myanmar ^27^. The number of dengue hemorrhagic fever (DHF) cases in Myanmar varies from year to year, with particularly high numbers in Yangon, Mandalay, and Ayeyarwaddy. More than 10,000 cases are recorded each year, causing on average 60 deaths ^28^. The number of dengue cases quadrupled between 1970 and 2015 ^27^. Outbreaks were recorded in 2009 (22,400 cases), 2013 (20,255 cases and at least 75 deaths), and 2015 (43,845 cases and 140 deaths) ^29^. A dengue outbreak occurred in 2017 with 31,000 cases and 192 deaths at the national level ^30^. In 2019, Myanmar also experienced an outbreak of chikungunya, with several cases recorded in travelers to the country ^31^. In Myanmar, there are more than 45,000 schools with eight million students, making up approximately 17% of the population ^32^. Most cases of dengue in Myanmar, occur in children under 15 years old, especially those in the 5–9-year age group ^33– 35^. One objective of the National Strategic Plan for Dengue Prevention and Control of Myanmar is to develop a comprehensive integrated training manual and guidelines including community and school-based vector control ^27^. This has led to the *Aedes*-free school program, which includes educational and practical activities.

Considering the global increase in dengue, effective dengue control should not rely on traditional insecticide-based, silver-bullet, top-down approaches ^36, 37^. Simple single-intervention approaches as practiced in the past were not successful. Suitable combinations of site-specific, effective, acceptable, and sustainable interventions need to be assessed for each situation ^38^. Therefore, we developed the current project for urban communities in Yangon, Myanmar. We aim to reduce dengue incidence and entomological risk factors, and improve knowledge, attitudes, and prevention practices by applying innovative combinations of school-driven non-insecticidal vector control tools, educational and knowledge transfer approaches by enhancing the school curriculum, building teachers’ capacity; and communicating behavioral change and transfer of knowledge following previous guidelines and recommendations ^25, 36, 37, 39^. A process evaluation will be conducted to supplement and strengthen the impact evaluation and to understand the processes and mechanisms of the interventions that can help explain impact or lack thereof.

## Methods

### Objectives and hypotheses

The overall objective of this trial is to assess the impact of student-driven interventions on dengue incidence, mosquito indices and dengue knowledge, attitudes and prevention practices in schools and communities.

The specific project objectives are to

1. Reduce dengue incidence in schools and communities.
2. Reduce entomological indices in schools and communities.
3. Improve knowledge of dengue, entomology, and vector control in students and their parents.
4. Engage students, teachers and relevant stakeholders in community vector control and monitoring.
5. Facilitate improved teaching capabilities by training of teachers and enhancing the dengue school curriculum.
6. Determine implementation fidelity and adaptation of interventions through process evaluation.

The main hypothesis of the study is that the dengue incidence rate will be lower in the intervention arm compared to the control arm. Other hypotheses are that mosquito indices will be lower; and that there will be improvements in the knowledge, attitudes and practices (KAP) regarding dengue risk reduction and community engagement in vector control activities in the intervention arm compared to the control arm.

### Trial design

This cluster randomized controlled trial (RCT) is designed to study the effect of integrated entomological and educational interventions on dengue incidence, adult dengue mosquito density and KAPs regarding dengue risk reduction among students and parents in two townships in Yangon, Myanmar. The rationale for a cluster design is that interventions are area-wide in character, since they will be implemented in schools, rather than targeting individuals. It is not feasible to randomize some individuals within a school to one intervention and other individuals to another. Schools (clusters) will be randomized into an intervention arm and a control arm.

### Study setting and participants

**Figure 1.**
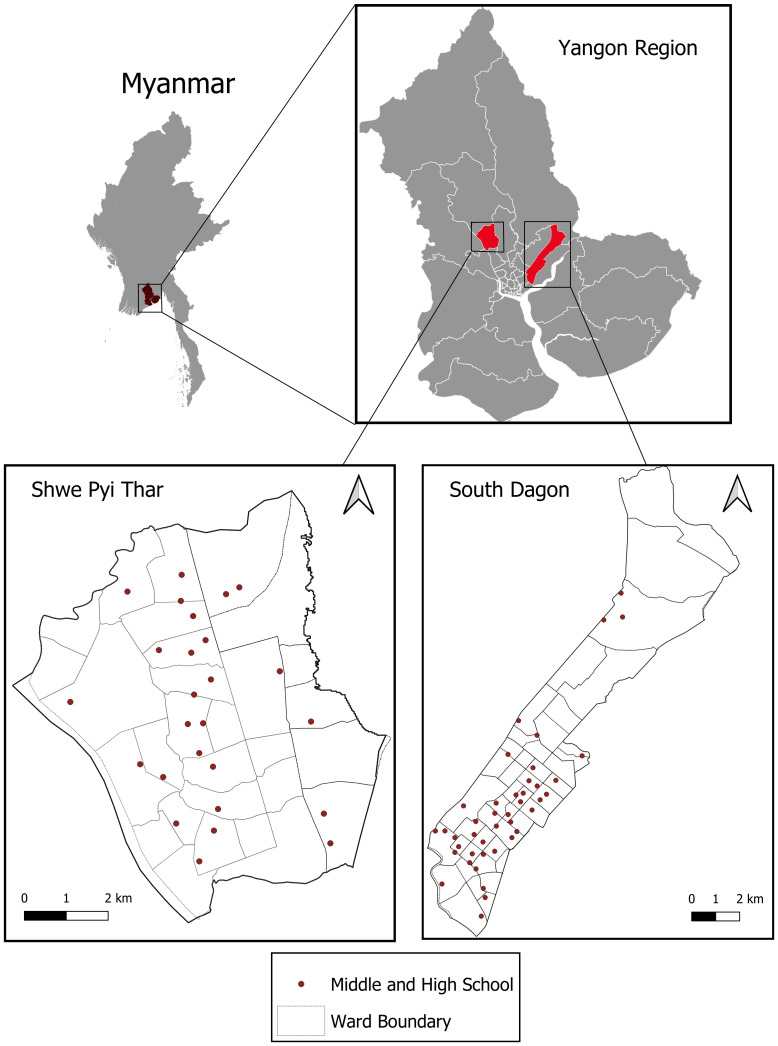
Map of study sites in Yangon, Myanmar. Data Source: Ministry of Education, Myanmar.

The study is planned to be conducted in South Dagon and Shwepyithar townships in Yangon Region, Myanmar ( Figure 1). These townships were selected out of the 45 in Yangon because of high dengue incidence and absence of other dengue projects. The population in South Dagon is ca. 307,000 and in Shwepyithar ca. 287,000. During 2013–2018 there were a total of 1,465 reported dengue cases (mean 244/year) and 15 deaths in South Dagon and 1,420 cases (237 cases/year) and eight deaths in Shwepyithar. These two townships represented approximately 12% of all reported dengue cases in Yangon Region during this period. There are 46 middle schools and high schools in these two townships, 20 in South Dagon and 26 in Shwepyithar. In 2019, there were an average of 250 sixth grade students per school. 40 high or middle schools will be included in the study ( Table 1). In each school approximately 40 sixth grade students (1–2 classes) will be selected based on feasibility, logistics, and willingness to participate. Each school and its catchment area (buffer zone) will be mapped. Non-overlapping school catchment areas that include students’ households represent the study clusters. Eligibility criteria are described in Table 1. It is anticipated that a minority of students (<10%) will live in a different school catchment area within the two study townships. Due to the current health and security situation in Myanmar, the project has been terminated by the donor, but the protocol forms a basis for future implementation in Myanmar or elsewhere.

**Table 1.** Eligibility criteria.

	Inclusion criteria	Exclusion criteria
**Schools**	- High and middle schools in selected townships in Yangon. - Schools that have at least 40 6 ^th^ grade students. - Approval by headmaster of school through signed informed consent and Letter of Agreement. - Approval by relevant teachers by signed informed teacher consent.	- Schools with other ongoing dengue interventions. - Schools with classes with fewer than 40 6 ^th^ grade students - International schools not using the national curriculum and Burmese as language of instruction. - Boarding schools.
**Students**	- Students who will start 6 ^th^ grade in June Year 1. - Signed informed assent. - Signed informed consent by parents.	- Students who are seriously ill and not considered physically fit to participate. - Students whose parents do not provide consent.

### Interventions

The interventions consist of integrated sets of vector control and educational/knowledge transfer interventions that will be implemented simultaneously. The justification for implementing sets of interventions instead of single interventions is the growing consensus that single interventions are not sufficient to control vector borne diseases because of the complexity of the transmission systems and their components ^37, 40^. Vector control tools must also be integrated with educational interventions and knowledge transfer to achieve sustainable change. Interventions will be implemented in schools and communities by students and teachers with project staff overseeing the quality and coverage of the interventions as part of the process evaluations (see below). Interventions will commence at the start of the school year (June Year 1). No interventions will be implemented in the control arm. However, schools and communities may receive control activities carried out by the national dengue control program. These schools also receive standard dengue control instructions from the School Health Division.

The vector control intervention has four components: (1) Adult mosquito mass trapping using commercial oviposition traps (Gravid Aedes Trap (BG-GAT) ^41^ and locally produced traps (plastic bottles) constructed by students will be placed in both schools and students’ households. Homemade traps will also be distributed to the communities by students. (2) Larval control using larvivorous guppy fish ( *Poecilia reticulata*). Guppy fish will be placed in suitable water storage containers in schools and households. Guppy fish production facilities will be set up in intervention schools, and students will learn how to manage these, propagate new fish, and distribute fish to their households and communities. (3) Prevention of mosquito breeding using tightly fitting lids or covers for water storage containers made of tarpaulin or other locally produced materials. (4) Source reduction by weekly solid waste management and cleanup campaigns by students around their schools and households.

The education and knowledge transfer intervention has two components: (1) Dengue curriculum enhancement and capacity building for teachers, consisting of a participatory approach to transformative education. Teachers and representatives from the Ministry of Health and Sports (MOHS) and the Ministry of Education (MOE), together with the research team, will review the current school curriculum and teacher’s manual, identify potential entry points, and co-create new teachers’ manuals, lesson plans, and activities in line with the project’s focus on participatory, inquiry and place-based education ^42, 43^. This approach can be deployed to solve community problems by engaging students and school staff and combined with an adapted Communication for Behavioral Impact (COMBI) ^25^ strategy will contribute to knowledge diffusion from schools to communities and improvement of health education outcomes. Activities will cover theoretical sessions on dengue disease, entomology, biology, vector control, and ecology combined with extra-curricular practices, such as field studies on mosquito ecology, breeding habitats, and mapping; mosquito reproductive biology (life cycle) experiments; adult mosquito trap creation; guppy-mosquito predator-prey interaction experiments; and larvae and adult surveillance. The enhanced dengue curriculum will subsequently be evaluated by teachers and adapted accordingly to ensure applicability and sustainability. (2) Communication for behavioral change and transfer of knowledge. The COMBI-approach ^25^ will be adapted to the current school settings, which will result in the development of key messages and communication materials through a school-community collaboration. Science fairs and dengue days will be organized once a year to provide a platform for hands-on student experiments and projects, display acquired knowledge, and encourage knowledge and perception sharing between students, teachers, parents, and the wider communities. During the fairs, exhibition of vector control techniques, student-led surveillance programs, and role-playing games involving students, parents, and teachers will be showcased to foster knowledge sharing and stimulate concerted actions for decentralized dengue surveillance and control. Community engagement will be carried out by students through school assignments and group work. Participatory epidemiology mapping activities and scenario building will be facilitated once per term where researchers, social science facilitators, teachers, and students collaborate on creating spatial representations of breeding sites and behaviors influencing transmission risk around the school areas. This will result in a map representing dengue epidemiological dynamics, as well as lead to the identification of a set of relevant, acceptable strategies for dengue prevention and control ^44^.

### Outcomes

The primary outcome is dengue incidence rates in communities ( Table 2)

**Table 2.** Primary and secondary outcome measures and other outcomes. Baseline is June when school starts in Myanmar. (Y1=Year 1, Y2=Year 2).

Outcome	No.	Name	Index	Description	Unit	Frequency of data collection	Details
Primary outcome	1	Dengue incidence rate in communities	DI	Confirmed number of dengue cases (numerator) and the estimated population in each school catchment area (denominator) during study period	No. cases during study period / population	Continuous	Passive case detection assessed using dengue rapid diagnosis tests (RDT) in public hospitals and health centers
Secondary outcomes	2	Seroconversion rates in school children	SR _S_	Seroconversion rates between two time points in students at school	No. of seroconversions / no. observation-days	3 times: - Jun. Year 1 (baseline), - Oct. Year 1, - Feb. Year 2.	Seroconversion rates assessed by dengue rapid diagnosis tests (RDT)
	3	Seroconversion rates in households	SR _H_	Seroconversion rates between two time points in 20 households in communities	No. of seroconversions / no. observation-days	3 times: - Jun. Year 1 (baseline), - Oct. Year 1, - Feb. Year 2.	Seroconversion rates assessed by dengue rapid diagnosis tests (RDT)
	4	Mosquito adult index in schools	AI _S_	Mean number of adult female *Ae.* *aegypti* and *Ae. albopictus* per school collected indoors and outdoors	No. / school	4 times: - Jun. Year 1 (baseline), - Oct. Year 1, - Feb. Year 2, - Jun. Year 2.	Mosquito collections using a battery- driven mechanical aspirator.
	5	Mosquito adult index in households	AI _H_	Number of adult female *Ae. aegypti* and *Ae. albopictus* per school or house collected indoors and outdoors	No. / household	4 times: - Jun. Year 1 (baseline), - Oct. Year 1, - Feb. Year 2, - Jun. Year 2.	Mosquito collections using a battery- driven mechanical aspirator.
	6	Breteau index in schools	BI _S_	No. of *Aedes* positive containers per 100 schools	No. / 100 schools	4 times: - Jun. Year 1 (baseline), - Oct. Year 1, - Feb. Year 2, - Jun. Year 2.	Mosquito collections in schools
	7	Breteau index in households	BI _H_	No. of *Aedes* positive containers per 100 houses	No. / 100 houses	4 times: - Jun. Year 1 (baseline), - Oct. Year 1, - Feb. Year 2, - Jun. Year 2.	Mosquito collections in households
	8	Knowledge, attitudes and practice scores in students	KAP _S_	Each of the KAP components measured in students	Sum scores on a scale from 1-10, where ten indicates the highest score	4 times: - Jun. Year 1 (baseline), - Oct. Year 1, - Feb. Year 2, - Jun. Year 2.	Determined using questionnaires
	9	Knowledge, attitudes and practice scores in parents	KAP _H_	Each of the KAP components measured in parents	Sum scores on a scale from 1-10, where ten indicates the highest score	4 times: - Jun. Year 1 (baseline), - Oct. Year 1, - Feb. Year 2, - Jun. Year 2.	Determined using questionnaires
	10	Behavioral assessment in students	BA _S_	Human decision-making in the uptake of vector control activities	Responses to statements on a 5-point bipolar-, or 5-point Likert scale	4 times: - Jun. Year 1 (baseline), - Oct. Year 1, - Feb. Year 2, - Jun. Year 2.	Data collected using questionnaires and structural equation modelling
	11	Behavioral assessment in parents	BA _H_	Human decision-making in the uptake of vector control activities	Responses to statements on a 5-point bipolar-, or 5-point Likert scale	4 times: - Jun. Year 1 (baseline), - Oct. Year 1, - Feb. Year 2, - Jun. Year 2.	Data collected using photovoice methodology and group discussions
	12	Engagement with students	E _S_	Degree of engagement in education/ knowledge transfer activities among students	Photographs and qualitative data	1 time after implementation of core interventions Nov. Year 1	Data collected using photovoice methodology and group discussions
	13	Engagement assessment with parents and teachers	E _PT_	Degree of engagement in education/ knowledge transfer activities among parents and teachers	Qualitative data and code book	Parents: 1 time: during parent-teacher meetings Nov. Year 1. Teachers: 3 times: - Jul. Year 1, - Nov. Year 1, - Mar. Year 2.	Data collected using qualitative methods: in-depth interviews and focus group discussions

The secondary outcomes are:

1) Dengue incidence rates in schools.
2) Mosquito adult index in schools and households.
3) Breteau index in schools and households.
4) Knowledge, attitudes and practice scores in students and parents.
5) Behavioral assessment of students and parents on dengue control and prevention.
6) Engagement assessment among students, parents and teachers in vector control activities.

### Sample size

The required sample size was estimated based on the primary outcome, dengue incidence in communities. Since the numbers of dengue cases in each school catchment area were unknown, the sample size is necessarily an estimate. This estimate was attained by using township level data from the two selected study townships. There was a total of 2,885 dengue cases in South Dagon and Shwepyithar townships during 2013–2018 giving an average of 480 cases/year during this period. Dividing the number of cases with the number of middle and high schools in these two townships gives a conservative estimate of 480/46=10.5 cases per school catchment area per year. We plan to recruit a minimum of about 40 students per school per year. The calculations would assume that in the control, the incidence is 10.5 dengue cases per 40 person-years of observation i.e., incidence rate of 0.26 dengue cases per person-year of observation and expect this to drop to approximately 0.18 dengue cases per person-year of observation in the intervention (i.e., 7.35 dengue cases per 40 person-years of observation). Using a cluster randomized design with 46 schools (23 schools per arm) and 10.5 cases per school catchment area at baseline, with α=0.05 this study has more than 80% power to detect an approximate 30% reduction in the dengue incidence rates between the control and the intervention arms. This estimate assumed an intra-cluster correlation coefficient (ICC) of 0.01. An intra-cluster correlation coefficient (ICC) for the dengue outcome is not available in the literature. We therefore used the next best available estimates, based on our previous assessment of ICCs for malaria prevalence and incidence in Myanmar which are low in this country, on average 0.003 (95% CI 0.000-0.010) ^45^. Although malaria is a different vector-borne disease with different transmission pattens in different settings, we think that the ICCs for malaria in Myanmar probably are the most realistic we can get and provide better estimates than unfounded guesses. The ICC is context-specific (location, spatial scale, etc.) and is preferred for studies with binary outcomes, such as this trial. As an additional guide, a reduction in dengue incidence by 25–30% would be consistent with the objectives of both the WHO Global Strategy for dengue prevention and control 2012–2020 ^46^ and the WHO Global Vector Control Response 2017–2030 ^37^. Lack of data on adult mosquitoes in the study area precluded a sample size calculation for the quantitative secondary outcomes on adult mosquitoes. However, the number of adult mosquitoes in the area is expected to be large ^47^, thus giving substantial power to detect differences between the two arms. All 46 schools will be included in the study, using an equal cluster size of 23 schools per arm. Both arms (intervention and control) will be represented in each township. The power calculations were performed in Stata 16.

### Recruitment

A series of project information, sensitization, and recruitment meetings will be held with relevant township administrators; school principals, teachers, and other school staff; and parents and students. Schools, teachers, students, and parents will be enrolled at these meetings. Participants will be informed that participating in the project is voluntary. Consent and assent procedures will be carried out during these meetings and letters of agreement between schools and the project signed. Additional meetings to inform healthcare workers in hospitals and health centers and training on how to collect patient data following Good Clinical Practice (GCP) guidelines will also be carried out.

### Randomization and blinding

Allocation of interventions will be done after baseline collections (described below). Of the 46 eligible schools, 23 will be allocated to the intervention arm and 23 to the control arm. This will be accomplished by an open public lottery event. At this event, representatives from each school will attend, including regional and township health and educational representatives, teachers, parents, and students. Information about dengue and the goals of the project will be given. The reasons for randomization, its procedures, and the concepts of intervention and control will be explained. Attendees will also have a chance to ask questions about dengue, vector control, health seeking behaviors, personal experiences of dengue, and specific details about the project. The lottery will be carried out as follows. Small pieces of paper, indistinguishable from one another, numbered from 1 to 46, will be folded and placed in non-transparent envelopes and mixed in a bowl. Each number represents a cluster (school) and a list of the schools with their respective number will be consulted during the process. A large screen with the numbered list of school names will be shown above the bowl, visible to everyone. A person not involved in the study, and accepted by all participants, will be selected to make the draw. Two flip boards with large sheets of paper will be placed on either side of the bowl with the heading ‘Intervention’ and ‘Control’ (in Burmese). The first number drawn (representing a specific school) will be assigned to the Intervention arm, the next number/school drawn will be assigned to the Control arm, and so on. Following the draw, the implications of being in either of the two arms will be discussed and the roles of participants, health volunteers, and sub-district hospital staff will be reviewed. By following this lottery scheme, the interventions are allocated at the same time as the sequence is generated, obviating the need for allocation concealment. At this meeting we will take care not to provide any information about planned dengue control interventions that will be used in the trial to participants from the control arm to minimize contamination between the groups. 

The study is unblinded for both participants and data collectors due to the nature of the intervention, e.g., it is not feasible to use placebo interventions of larvivorous fish.

### Data collection

**Figure 2.**
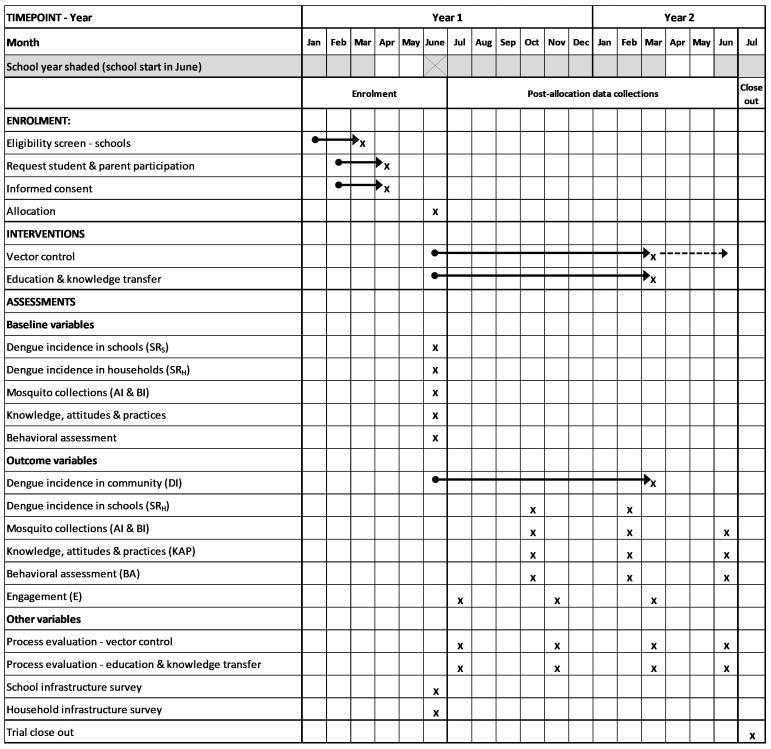
Time schedule of enrolment, interventions, and pre- and post-allocation data collections based on Standard Protocol Items: Recommendations for Interventional Trials (SPIRIT) checklist ^48^. Arrows indicate continuous activities. Dashed arrow indicates that some of the vector control interventions will continue until June Year 2. Primary and secondary outcome measures in parentheses; see Table 2 for further details.

***Disease surveillance.*** The primary outcome will be assessed by passive case detection in communities and in schools. The number of dengue cases in communities will be assessed during the study period ( Figure 2) through all hospitals and health centers in the two townships. These will be supported by monthly visits and provision of rapid diagnostic test (RDT) kits by the project. Cases of fever in participating schools will be monitored during school hours. In both communities and schools, fever cases with dengue-like illness fitting the WHO case definitions using clinical symptoms (fever, severe joint and muscle pain, intense headache, rash, etc.) will be recorded and RDTs performed using an antibody/antigen RDT (SD BIOLINE Dengue Duo NS1/IgG/IgM test kit, Standard Diagnostic Inc., Korea). Students and staff in schools will be referred to a nearby participating hospital, clinic, or school health medical offices for confirmation using RDTs as above. Written informed consent will be taken from each person who agrees to participate in the study. The RDT cassette will be used for confirmation of dengue virus and serotype using polymerase chain reaction (PCR).

The secondary disease outcomes will be assessed by seroprevalence assessments in schools and households at baseline (June Year 1) and at two follow-ups (four and eight months after baseline) and seroconversions between these two time points. In each school, a total of 40 students, teachers and staff (20 randomly selected 6 ^th^ grade students, 20 randomly selected students in grade 1–5 based on feasibility and financial reasons) will be asked to provide a finger prick blood sample for a dengue RDT. Similarly, seroprevalence will be assessed in 40 households per school catchment area of which 20 will be students’ households and 20 randomly selected households in each school catchment area. One randomly selected person (any age >1 year old) in each household will be selected. Thus, there will be 20*23 = 460 individuals in each group (6 graders, grades 1–5, student households and other households) which permits detection of a 30% difference in seroconversion between the two study arms allowing for a 10% dropout rate, with 80% power and 5% significance. The presence of dengue antibodies (IgG and IgM) will be confirmed by a finger prick blood sample of 10μL and a rapid diagnosis test (RDT) kit (SD BIOLINE Dengue IgG/IgM, Standard Diagnostic Inc., Korea). A seroconversion will be determined when an RDT negative person becomes positive in a subsequent collection. Written informed consent will be taken. Data on potential confounders, e.g., age, sex, residence, travel history, and previous dengue history will be collected for each person using a short case report form (CRF).

***Mosquito collections.*** Mosquitoes will be collected in each of the selected 46 schools and in at least five of the 20 selected households per school at baseline (June Year 1) and at three follow-ups (four, eight, and 12 months after baseline). In each school, adult mosquitoes will be collected using battery-driven aspirators for 15 minutes indoors (classrooms, canteen, toilets) and 15 minutes outdoors (at typical student gathering points, e.g., in vegetation). In each school, all breeding sites will be inspected and the following recorded: number of total containers, number of wet containers, number of containers positive for mosquito immatures (all genera and species), container type, and location (indoors/ outdoors). Mosquito larvae will be collected from all positive containers using regular sweep nets, dippers or strainers. Pupae will be collected using the pupal-demographic survey method ^5^ and the single water-surface sweep-net procedure for large containers ^49^. Mosquito collections in households will be done in the same way, except that adult mosquitoes will be aspirated for 10 minutes indoors (living room, bedroom, toilet, kitchen) and 10 minutes outdoors (e.g., in vegetation). In addition, entomological monitoring, one continuous week each month, will be done using BG-GAT ovitraps (glue strips) in both schools and households and. Traps will be managed by students as part of the enhanced school health curriculum and hands-on activities and will be compared with data collected by project teams. After the one week, mosquitoes on glue strips will be collected by project staff and identified. Species composition of mosquito larvae will be determined ( *Ae. aegypti, Ae. albopictus, Culex, Anopheles,* and ‘Others’). *Aedes* pupae will be identified and sexed using published keys ^50^. Adult mosquitoes will be sorted by sex and identified to species level ( *Ae. aegypti, Ae. albopictus, Culex* spp. and ‘Others’) using a stereomicroscope. Presence of blood and blood digestion status (Sella’s stages) of female mosquitoes will be determined by external examination of abdomens. Dissection will be done in a sample of *Ae. aegypti* and *Ae albopictus* to assess their parity status. A commercially available DENV NS1 ELISA kit, the Platelia Dengue NS1 Ag kit (Bio-Rad Laboratories; Catalogue no. 72830), will be used to detect DENV in a sample of collected mosquitoes. Heads and thoraces of individual adult mosquitoes will be stored separately in 1.5 mL Eppendorf tubes. The abdomens will be pooled (five-10 mosquitoes per pool depending on catches) and stored in 1.5 mL Eppendorf tubes. Mosquitoes will be stored in -20°C. Virus detection will be performed on pooled and individual specimens using real-time RT-PCR (qPCR) and primers and probes targeting all four DENV serotypes ^51^. Positive samples will be submitted to a second run specific qPCR to determine the DENV serotype ^52^. 

***Knowledge, attitudes, and practices (KAP).*** KAP measurements regarding dengue and vector control in students, parents, and teachers, will be assessed using pretested surveys on electronic forms on computer tablets. Endpoint sum KAP scores on a scale from 1–10, where 10 indicates the highest score, will be calculated for students and parents. KAP surveys will be done at baseline (June Year 1) and at three follow-ups (four, eight, and 12 months after baseline). Additional methods to assess changes in KAP and behaviors include key informant interviews, focus group discussions, in-depth interviews, and observations. Data for the behavioral models (below) will be collected through validated constructs included in the KAP surveys.

***Behavioral assessment.*** Despite the widespread application of KAP measurements, a common limitation is that these assume all behaviors to be rational and requiring a high level of cognitive effort. KAP measurements fail to take into account irrational determinants of human behavior such as heuristics (i.e., adaptive-thinking approaches when making decisions in complex situations) and cognitive biases (i.e., cognitive pitfall where people make systematic reasoning errors resulting from reliance on mental shortcuts) stemming from behavioral economics. Behavioral economics is a discipline combining both economics and psychology, which aims to provide an alternative perspective to the assumption that behavior is governed by rational decision making, as exemplified in traditional economics ^53^. The behavioral assessment will be conducted to include relevant factors (e.g., heuristics, biases) influencing dengue prevention, in addition to the factors captured by the KAP measurement. These factors will be identified through the literature and formative research. Results from the behavioral assessment will be used to develop a more comprehensive model to understand and predict people’s behavior in relation to dengue control and prevention. Results and models will be compared between intervention and control arms and between pre- and post- intervention groups. This will allow estimation of direct and indirect effects of the interventions on vector control behaviors. Data for the models will be based on validated constructs, representing the identified behavioral factors, included in the KAP surveys. Constructs are abstractions that are created and validated by researchers in order to conceptualize a latent variable, which is correlated with scores on a given measure, although not directly observable. The scores of each measure will be recorded on a six-point bipolar scale (e.g., good-bad), or on a six-point Likert scale in which the respondents will present their opinion on a scale ranging from strongly disagree to strongly agree and adjusted for children’s cognitive skills ^54, 55^. Structural equation modeling will be used to validate and measure the model parameters (see data analysis).

***Engagement assessment.*** Degree of engagement among students, parents, and teachers in vector control activities will be assessed using a set of qualitative methods, including photovoice ^56^, in-depth interviews, and focus group discussions. In the photovoice sub-study students will take photographs that reflect their experiences and perspectives, engaging them in dialogue on community health issues and reaching influencers and key stakeholders to catalyze social and behavioral change. Thus, students will develop an understanding of perspectives and understanding of mosquitoes, vector control, and any behavioral changes in relation to vector control activities, including unanticipated changes or consequences as a result of participation in the intervention. A group of approximately 10 students in four intervention schools, two in each township, will be invited to participate. Students will be trained in how to use cameras and on ethical issues associated with taking photos, and subsequently asked to generate photo assignments related to dengue and the dengue control interventions. A facilitator will moderate group discussions with participants whereby ‘trigger’ photographs are selected through a structured activity using a Freirean-based inductive questioning technique ^57^ used to catalyze critical dialogue to encourage reflection and generate new understanding of the issue students choose to highlight in their photographs. Teachers’ engagement will be assessed through in-depth interviews (approximately 23 teachers both pre- and post-intervention to include all intervention schools). Additionally, there will be two focus group discussions conducted post-intervention. With teachers, the following topics will be discussed: 1) roles and responsibilities in vector control education/activities; 2) school outreach activities; and 3) operational or social challenges of education and knowledge transfer to the wider community. Parental engagement will be assessed using in-depth interviews held during parent-teacher meetings (approximately 46 parents, two parents per intervention school). This will gauge the effect of the intervention on bi-directional knowledge flow and engagement between schools, households and the wider community. In particular, interview guides for parents will explore five areas relating to community engagement and the social context of the education and knowledge transfer interventions: 1) personal history (migration, poverty, conflict); 2) perceived health problems (dengue/fever concepts); 3) perceptions of vector control activities; 4) involvement in the school outreach interventions; and 5) perspectives and experiences related to community engagement for vector control and prevention throughout the whole intervention ^58^.

***Process evaluation.*** A systematic process evaluation in the intervention arm will be conducted to assess implementation fidelity and adaptation, as well as perception of the dengue control interventions. Implementation fidelity, i.e., the extent to which the intervention was implemented as intended will be assessed, as will adaptation, i.e., the extent to which participants introduce changes to the original components/activities of the intervention to address contextual needs and challenges ^59^. The conceptual framework for implementation fidelity developed by Carroll *et al.* ^60^ will be applied using specific process evaluation measures ( Table 3). Implementation fidelity will be assessed using an index score (ranging from 1 to 10) comprising variables that assess intervention exposure, dose, adherence, quality of delivery, program adaptation, and responsiveness. Index score cards will be developed for both the vector control and education/knowledge transfer interventions. The vector control interventions will be assessed through observation in school premises and households during four visits after implementation of the interventions ( Figure 2). The first three visits will be performed during the trial year, a follow-up visit will measure sustainability of the vector control activities. The index score cards for measuring vector control fidelity will include measurements of lid coverage, guppy fish and trap maintenance and waste management.

**Table 3.** Process evaluation measures.

Measure	Description	Unit	Frequency of data collection	Details
Implementation fidelity of the vector control interventions	The extent to which the vector control activities were implemented as intended at schools and in households	Sum scores on a scale from 1–10, where ten indicates the highest score	4 times: - Jul. Year 1, - Nov. Year 1, - Mar. Year 2, - Jun. Year 2.	Assessed by observation and using index scorecards
Implementation fidelity of the education/knowledge transfer interventions	The extent to which the education/ knowledge transfer activities were implemented as intended by teachers	Sum scores on a scale from 1–10, where ten indicates the highest score, logbooks and qualitative data	4 times: - Jul. Year 1, - Nov. Year 1, - Mar. Year 2, - Jun. Year 2.	Assessed by observation and using index scorecards, as well as through logbook data collection
Reflection and adaptation of the intervention components	To develop an understanding of perceptions and experiences related to the education/ knowledge transfer interventions	Logbooks, qualitative data and code book.	2 times: - Nov. Year 1, - Mar. Year 2,	Assessed using teachers’ logbooks and qualitative methods: in-depth interviews and focus group discussions

The education and knowledge transfer interventions will be measured through observation using video recordings as to minimize social desirability and observer bias. The recordings will take place at four key time points: first when MOE and MOHS representatives deliver the enhanced curriculum to the teachers; then two times in the school context, when teachers deliver the curriculum to the students; and finally, a follow-up measurement of sustainability and adaptation, when the representatives of the MOE and MOHS deliver the curriculum to a new group of teachers for the upcoming schoolyear. As described above, the curriculum, and associated teachers’ manuals, will be developed through a participatory approach which will result in the development of the core components of the education/knowledge transfer intervention. Accordingly, the instruments to measure implementation fidelity of these interventions will be developed based on these core components and included in the index score card. The score card scores will be compiled into a comprehensive implementation fidelity index, similar to what has been used in water, sanitation and hygiene interventions ^61^. Teachers will be trained in using a logbook for documenting their experiences with the curriculum and the manual, to be able to record their own implementation fidelity and adaptation from the educational/knowledge transfer interventions. The logbook assessment will focus on the dynamics of the educational interventions, the teachers’ roles, adaptation to changes brought about by activities, implementation fidelity moderating factors (i.e., comprehensiveness of policy description, strategies to facilitate implementation, quality of delivery, and participant responsiveness), barriers, and facilitators of the implementation, and any unintended effects ^62^. Notes from the logbooks will be compiled into a manual that will serve as adaptation guidelines for the following years.

In addition, focus group discussions and in-depth interviews with parents and students will be conducted to develop an understanding of perceptions and experiences related to the education/ knowledge transfer intervention. The total number of discussions will be guided by the principle of data saturation, but there will be at least five focus groups (during two time points) with parents and students, respectively; and 20 in-depth interviews to elicit more nuanced data on emerging themes stemming from the focus groups. Qualitative data will be transcribed in the language in which it was conducted, translated into English and back translated to ensure accuracy. Findings from the quantitative and qualitative data will be triangulated to provide a comprehensive overview of the implementation process.

***School and household surveys.*** School infrastructure surveys will be conducted at baseline in all schools to determine number of students, teaching and non-teaching staffing capacity personnel, existing health activities, facilities (e.g., classrooms, water and sanitation and hygiene facilities). A household survey will be conducted in all households of the participating students. It comprises a household questionnaire with the number of people living in the house (household roster), and their age, sex, level of education, occupation, and whether each inhabitant is a permanent member of the household. In addition, data will be collected on room occupancy, household assets, socioeconomic status, type and quality of house structure (roof, walls, ceilings, and floors), sanitation facilities, household water management practices, source of drinking and non-drinking water, solid waste collection and disposal, hygiene practices domestic animals, and mosquito control activities. The household roster will be updated at each entomological data visit. The geographical coordinates of each school, participating student’s households and school catchment areas will be mapped using handheld Global Navigation Satellite System (GNSS) devices and satellite imagery combined in a Geographical Information Systems (GIS).

***Climate and environment.*** Data on rainfall, temperature, and humidity will be collected by measurements in schools, as well as from nearby meteorological stations. Household densities (crowding) in school surroundings and school catchment areas will be collected from annotation of satellite images, as will land use including the presence of non-residential areas, vegetation, and water bodies.******

### Data management

Unique identification numbers will be given to each participating school, teacher, student, and household. Each household member will also receive a project ID number. Data will generally be collected using pre-programmed tablets. A detailed Data Management Plan (DMP) has been developed using ‘FAIR’ principles for Horizon 2020 projects (i.e., data are Findable, Accessible, Interoperable and Re-usable) ^63^. Data will be stored in a password protected, anonymized database, which will only be accessible by the study team. Any documentation containing information about patients or biological specimens will be kept in a locked cupboard at the Malaria Consortium office in Yangon. Automated data quality checks will be built into the data collection system with further checks at the time of data extraction and analysis. Spatial data on household locations, mosquito collection sites, and climate will be stored in a spatial geodatabase. This will be supported by a project data manager based in Yangon and supported by the MORU Clinical Trials Support Group and Epidemiology Departments in Bangkok.

### Data monitoring committee

The formation of a data monitoring committee was assessed not to be required, because the trial is considered low risk for participants and of short duration. For the same reasons interim analyses and stopping guidelines were not included in the study.

### Adverse events monitoring and reporting (harms)

No enhanced risks are expected for the participants in this project, whether physical, mental, or social. There is no compensation for study participants (including expenses and access to medical care).

The proposed interventions are environmentally friendly and do not include drugs, vaccines, insecticides, or other chemical substances. Certified nurses, doctors, technicians, or trained project staff will used single-use sterile safety lancets to take blood samples from participants. These lancets are designed to minimize pain during blood sampling and reduce the risk of needle injury by immediate retraction of the needle after use. The interventions are therefore considered safe for humans and have no negative consequences for the external environment. International concerns have been raised whether release of guppy fish might escape into the environment and negatively affect native biodiversity ^64^. In this project, guppies will be distributed to isolated water storage containers and are not likely to escape into the environment. There are few, if any, studies showing that the risk of guppy escape into local ecosystems as a result of vector control is high and detrimental to aquatic ecosystems. Furthermore, the National Guidelines for Dengue Prevention and Control in Myanmar recommend the use of larvivorous fish as a dengue larvae control strategy ^65^.

### Auditing

There will be no formal auditing of this trial.

### Data analyses

***Effect of intervention on primary outcome***

1. **Dengue incidence rate in communities (DI).** The total cumulative number of dengue cases in the communities will be recorded passively through patients presenting at collaborating public hospitals and health centers in the two townships. Each case will be confirmed by dengue antigen/antibody RDTs. Each case that resides in the school catchment areas will be noted as such. The denominator will be the estimated population in each school catchment area. Data analysis for the primary outcome will be a comparison of the overall difference in incidence rate of confirmed dengue cases between the intervention and non-intervention school catchment areas with sub-analyses comparing the monthly incidence over time, before, during and after the interventions to examine the timing of any intervention impact. Incidence data will be collected continuously over a one-year period. At the cluster level, analysis will be by intention to treat, i.e., taking the trial arm as that to which, each cluster was randomized. At the individual level, people will be taken to have the allocation of the arm in which they are resident at the time of any data contributed. A flowchart showing numbers of clusters, and average numbers of households per cluster, over time will be constructed in accordance with Consolidated Standards of Reporting Trials (CONSORT) guidelines ^66^. For the primary analysis, missing data may occur if complete clusters decline to continue in the trial. In this case, the cluster will still be included as long as any data on the primary outcome are available. This does introduce a risk of bias in estimating effectiveness, if loss of clusters is related to performance of the interventions. Dengue incidence in communities will be analyzed using a Poisson regression model. A negative binomial regression will be considered if there will be over-dispersion in the Poisson distribution. The response variable will be the number of dengue cases per cluster and the exposure will be the person-time at risk. Hence, the analysis will yield rate ratios. Multilevel models will not be used for this outcome since the number of cases may be too small for them to be fitted robustly. Statistical significance will be declared at 5% significance level. The 95% confidence intervals will be calculated and reported where applicable. A detailed statistical analysis plan (SAP) will be developed prior to database lock.

***Effect of intervention on secondary outcomes***

2. **Seroconversion rates in schools (SR _S_).** The proportion of dengue antibody seroconversion in schools will be calculated as the number of seroconversions between the baseline and subsequent follow-ups at four and eight months after the implementation of the interventions divided by observation-days of school populations. The definition of seroconversion is when a participant has converted from being IgG (or IgM) negative during one sampling event to being IgG (or IgM) positive during a second sampling event. Dengue incidence based on seroconversions will be analyzed using a Poisson regression model. A negative binomial regression will be considered if there will be over-dispersion in the Poisson distribution. The response variable will be the number of dengue cases per cluster and the exposure will be the person-time at risk. Hence, the analysis will yield rate ratios. Multilevel models will not be used for this outcome since the number of cases may be too small for them to be fitted robustly.
3. **Seroconversion rates in households (SR _H_).** The proportion of dengue antibody seroconversion incidence in a selection of 40 households per school catchment area will be calculated and analyzed in the same way as for schools.
4. **Mosquito Adult Index in schools (AI _S_).** This the number of adult female *Ae. aegypti* and *Ae. albopictus* (combined) per school or per house collected both indoors and outdoors for 15 minutes at each location (30 min total collection time) using a battery-driven mechanical aspirator. Collections will occur at baseline and once every four months in all schools. A negative binomial regression will be done with the number of mosquitoes as the outcome variable and number of school-visits as the exposure (denominator) variable, i.e., the logarithm of the number of schools as the ‘offset’. A logarithmic link function will be used. Hence, the exponential of the coefficient for arm will be the between-arm ratio in Adult Index (AI) according to the response variable used. Additional analyses of the AI _S_ will be done by including the baseline value as an additional covariate. Due to the potential skewness of these values, this will be done by categorizing the index as zero, or above or below the median of the positive values.
5. **Mosquito Adult Index in households (AI _H_).** This is the same as AI _S_ except that collections in households will be done for 10 minutes indoors and outdoors, respectively (20 min total collection time). Collections will occur at baseline and once every four months in five selected households per cluster. This index will be calculated and analyzed in the same way as AI _S_.
6. **Breteau Index in schools (BI _S_).** The BI is the number of containers with *Aedes* immatures/100 schools. It will be calculated at baseline as well as for each of the three follow up time points. This index will be analyzed similarly to the Adult Index.
7. **Breteau Index in households (BI _H_).** This BI is the number of containers with *Aedes* immatures/100 houses. It will be calculated at baseline as well as for each of the three follow up time points. This index will be analyzed similarly to the Adult Index.
8. **Knowledge, attitudes and practice scores in students (KAP _S_).** These are based on sum scores on a scale from 1–10, where ten indicates the highest score. Scores will be calculated at baseline and at each of the three follow up time points. The total score for each of the three KAP components will be added, and the resulting value expressed on a scale from 0 to 10. The average KAP scores for each school will be calculated over all surveys done in the intervention period. These school-level averages will serve as the response variable for a statistical analysis using linear regression. The explanatory variable will be whether or not the school received the intervention.
9. **Knowledge, attitudes and practice scores in parents (KAP _H_).** These scores will be calculated at baseline as well as for each of the follow up time points and analyzed in the same way as explained above.
10. **Behavioral assessments (BA _S_ and BA _H_).** A behavioral model will be developed based on the latent variables collected through the survey and validated with structural equation modeling. The model will be evaluated, and a model fit determined, using the following indices: the Comparative Fit Index (CFI) and the Tucker Lewis Index (TLI) (CFI/TLI > 0.90), the Root Mean Square of Approximation (RMSEA) (<0.08) and the Standard Root Mean Square Residual (SRMR) (<0.10) ^67^. Two multi-group analyses will be performed using two control variables: control vs. intervention arm and pre- vs. post- intervention. To do so, measurement invariance will be tested and compared through Chen’s cut-off points for the Comparative Fit Index (CFI) and McDonald’s Non-Centrality Index (NCI) ^68^. To test change in fit between nested models suggested thresholds of 0.005 for ΔCFI and 0.010 for ΔNCI will be used
11. **Engagement assessment with students (E _S_).** The photovoice sub-study contribute to developing an understanding of students’ degree of engagement and perceptions and experiences of being a participant in the intervention through photographs as well as qualitative data including photo discussion sessions. The approach to data analysis will be inductive and include thematic analysis of the transcribed photo discussion sessions involving the students as co-researchers through member checking of the code book to ensure inter-coder reliability. This process is often used to assess the trustworthiness of qualitative research findings ^69^.
12. **Engagement assessment with parents and teachers (E _PT_).** The degree of engagement in interventions in parents and teachers will be assessed using qualitative methods. The approach to data analysis will combine inductive and deductive elements, using determinants and typologies of community engagement ^70, 71^. Analytical categories will be developed from the initial research questions and also emerge during the analysis process. Using NVivo, identified categories will be operationalized as codes in a flexible coding scheme. The content of the codes will be discussed extensively between independent coders, and subsequently used to develop themes and to explore patterns.

### Process evaluation

**Implementation fidelity scores** for both the vector control interventions and the education/knowledge transfer interventions will range from 1–10, where 10 indicates the highest score when implementation of the interventions were executed as intended. The qualitative data collected from teachers’ self-report logbooks will be analyzed using an inductive approach. Principal component analysis will be conducted using implementation fidelity data and outcomes performance from different implementation units of the intervention to estimate the impact of intervention variability on the results.
**Reflection and adaptation.** Qualitative data from in-depth interviews and focus group discussions will be analyzed using an inductive approach. In addition, feedback sessions with participants including teachers, parents and students will be held to share the code book of the themes generated during analysis to ensure a participatory process, as well as to increase the accuracy and enhance the quality of the data analysis.

## Discussion

The global increase in dengue during many decades and the difficulties to control the disease are likely caused by multi-factor determinants in complicated relationships that differ from location to location, depending on physical, environmental, socioeconomic, behavioral and cultural factors. Since traditional insecticide-based or single-intervention approaches have not appeared to be successful, we think it is important to test and assess different combinations of hard (physical) and soft (behavioral, educational) interventions that are unique to specific locations. Even though new vector control interventions have been developed and showing positive results, such as the release of Wolbachia-infected mosquitos ^72^, success will likely be higher if integrated with soft interventions. Such approaches would harmonize with international vector-borne disease control frameworks and recommendations ^12, 36, 37, 39, 46^. In order to assess how the interventions were implemented we will implement comprehensive process evaluation and implementation fidelity approaches. This will allow detection of potential weak points in the implementation process.

This trial will evaluate a novel combination of vector control and educational interventions to control dengue in urban settings in Myanmar. The trial differs from many other dengue control trials in that it combines several unique vector control interventions combined with educational and communication approaches in a school-community context. It focuses on vector control tools that do not rely on insecticides to which mosquitoes will eventually develop resistance and thus render the intervention unsustainable. The combination of vector control interventions targets both adult and immature stages of the mosquito aiming at reducing oviposition through container covers and clean up campaigns, increasing immature predation by larvivorous fish, and trapping adult mosquitoes using mass trapping designs. Much research is currently ongoing to develop sustainable mosquito mass trapping designs as viable options for vector control ^73^.

Disease control interventions should be integrated and anchored in community processes, where schools could play important roles by engaging students, parents and teachers. By improving and/or adjusting school health curricula to include vector and disease control sessions, knowledge and behavioral change could be disseminated to communities by students ^18, 74^. Knowledge, attitudes, and self-efficacy of students for dengue prevention and control can be improved by using educational games, cartoons, observations, lectures and information campaigns ^18^. Since there is a lack of randomized controlled trials to assess the effect of school-based educational interventions on dengue and risk behaviors ^18^, our trial would contribute to this gap in knowledge. Health authorities in Myanmar are developing integrated training manuals and guidelines for community and school-based vector control, including the *Aedes*-free school program ^27^.

Dengue transmission dynamics exhibit strong spatial and temporal variations. Therefore, it is always difficult to assess the effect of dengue control interventions as they may fall in years with low and insufficient number of cases. Although we cannot predict future outbreak patterns, we have selected townships with the highest number of cases during the preceding years and where no other dengue interventions have been implemented. A longer intervention period would increase the likelihood of an outbreak year and avoid interepidemic periods, however, this will also reflect in costs and feasibility constraints. Another, limitation inherent in dengue research is the presence of relatively high proportions of asymptomatic cases, who, of course, will not show up in clinics and in national statistics.

### Ethics policies

This trial is registered as ISRCTN78254298 in the ISRCTN Registry on 31 May 2022, https://www.isrctn.com/ISRCTN78254298. The trial was approved by the Institutional Review Board 1, Office no. 4, NayPyiTaw, Union of Myanmar, (IRB 1/ 2019-7; 12/06/2020) and the Regional Committee for Medical and Health Research Ethics, Section B, South-East Norway (REK) (2019/814/REK sør-øst C; 26/06/2019). Any protocol amendments will be submitted to these committees and the ISRCTN trial registry will be updated. All trial participants will be engaged in a process of documented informed consent and assent before participating in any study activities. Project staff and MOHS staff trained in informed consent procedures will request participants to give informed consent to take part in the study, and in case of children, informed assent in addition to consent from parents or guardians. Preliminary sensitization meetings will identify potential schools, principals and teachers that are positive in participating in the project, therefore avoiding issues with principal and teacher dissent. Potential problems with dissent by students or student parents can be avoided since the 6 ^th^ grade student population is so large that there are sufficient classes and students to enroll in the project. There are six written informed consent procedures (can be requested from corresponding author): 1) Consent for school participation by school headmaster, 2) consent from teachers, 3) assent from students, 4) consent from parents, 5) consent for requesting a blood sample (taken each time a blood sample is required), signed by participants aged 18 years or older and by legal representative, parent or guardian if participant is 10 years or younger, and 6) informed assent for requesting a blood sample (taken each time a blood sample is required) signed by participants aged 10–17 years. All consent and assent forms explain the risks and benefits of the study. For participants who cannot read, the entire informed consent form will be read and explained by the project staff in the presence of a community witness. After consenting, these people will mark an inked thumb impression on the form, and the witness will be asked to sign it. Consent and assent forms approved and stamped by the MOHS are used in accordance with the ethics committees’ guidelines. Project staff will follow the standard operating procedures (SOPs) developed in the project regarding data collections and guides to obtain consent. All consent forms and project information sheets will be provided in Myanmar language. Participants will be given enough time to ask for clarification and consider if they wish to participate, and they will be offered to have the information read out to them if they prefer. Participants who cannot read or write will consent by a thumbprint. Participants less than 10 years old will not sign a consent or assent form, but parents or guardians will sign the consent form on their behalf. An age-appropriate project information sheet is available for young participants. Participants will keep the information sheet, while the researchers will keep the signed consent forms. Participants have the right to withdraw from the study before, during or after the data collection activity, up to the completion of the report summarizing the analysis of the data collected. This manuscript contains no individual person’s data, so consent for publication is not required.

### Confidentiality

To ensure participants’ confidentiality, a code will be assigned to each participant, which will be used instead of individuals’ names on data collection tools, in transcripts, reports and field diaries. No information that can be used to identify individuals will be transcribed or reported. An electronic master list of names and codes will be kept, which will be filed in a restricted location on Malaria Consortium’s intranet. Hard copies of consent forms or attendee lists will be kept in a secure, locked location in the Malaria Consortium office in Yangon. All confidential materials will be accessible to senior members of the study team. No files relating to the activity will be left on the laptops or other devices used by the temporary researchers or the audio-recorders. Audio-recordings will be deleted once reports summarizing the findings have been finalized. The data custodian for data related to each work package will be the heads of work packages. All data collection activities related to the social and KAP components will be conducted in safe and private spaces, ensuring that, as much as possible, participants cannot be overheard by non-participants. The KAP surveys will be conducted in schools and at participants’ households. Data collected on cases with fever presenting to health facilities and tested for dengue will be anonymized.

### Ancillary and post-trial care

The trial is deemed minimal risk to study participants. Therefore, there are no provisions for ancillary or post-trial care, or for compensation to those who suffer harms from trial participation, beyond the existing private or social security system. The original project sponsor (NMBU) had a specialist insurance policy which would operate in the event of any participant suffering harm as a result of their involvement in the research.

## Data availability

No data are associated with this article.

## References

[1] Bhatt S Gething PW Brady OJ : The global distribution and burden of dengue. *Nature.* 2013;496(7446):504–507. 10.1038/nature12060 23563266 PMC3651993

[2] WHO: Dengue - Guidelines for diagnosis, treatment, prevention and control.In: Geneva; 2009. Reference Source 23762963

[3] Gubler DJ Halstead SB : Is Dengvaxia a useful vaccine for dengue endemic areas? *BMJ.* 2019;367:l5710. 10.1136/bmj.l5710 31582375

[4] Low JG Ooi EE Vasudevan SG : Current Status of Dengue Therapeutics Research and Development. *J Infect Dis.* 2017;215(suppl_2):S96–s102. 10.1093/infdis/jiw423 28403438 PMC5388029

[5] Focks DA : A review of entomological sampling methods and indicators for dengue vectors.In: Geneva; 2003;38. Reference Source

[6] Bisanzio D Dzul-Manzanilla F Gomez-Dantés H : Spatio-temporal coherence of dengue, chikungunya and Zika outbreaks in Merida, Mexico. *PLoS Negl Trop Dis.* 2018;12(3):e0006298. 10.1371/journal.pntd.0006298 29543910 PMC5870998

[7] Stanaway JD Shepard DS Undurraga EA : The global burden of dengue: an analysis from the Global Burden of Disease Study 2013. *Lancet Infect Dis.* 2016;16(6):712–723. 10.1016/S1473-3099(16)00026-8 26874619 PMC5012511

[8] Brady OJ Gething PW Bhatt S : Refining the global spatial limits of dengue virus transmission by evidence-based consensus. *PLoS Negl Trop Dis.* 2012;6(8):e1760. 10.1371/journal.pntd.0001760 22880140 PMC3413714

[9] Spiegel J Bennett S Hattersley L : Barriers and bridges to prevention and control of dengue: The need for a social–ecological approach. *Ecohealth.* 2005;2:273–290. 10.1007/s10393-005-8388-x

[10] Heintze C Velasco Garrido M Kroeger A : What do community-based dengue control programmes achieve? A systematic review of published evaluations. *Trans R Soc Trop Med Hyg.* 2007;101(4):317–325. 10.1016/j.trstmh.2006.08.007 17084427

[11] Gubler DJ : Dengue, Urbanization and Globalization: The Unholy Trinity of the 21(st) Century. *Trop Med Health.* 2011;39(Suppl 4):3–11. 10.2149/tmh.2011-S05 22500131 PMC3317603

[12] Dusfour I Vontas J David JP : Management of insecticide resistance in the major Aedes vectors of arboviruses: Advances and challenges. *PLoS Negl Trop Dis.* 2019;13(10):e0007615. 10.1371/journal.pntd.0007615 31600206 PMC6786541

[13] Wilcox BA Aguirre AA De Paula N : Operationalizing One Health Employing Social-Ecological Systems Theory: Lessons From the Greater Mekong Sub-region. *Front Public Health.* 2019;7:85. 10.3389/fpubh.2019.00085 31192179 PMC6547168

[14] García-Rejón J Loroño-Pino MA Farfán-Ale JA : Mosquito infestation and dengue virus infection in *Aedes aegypti* females in schools in Merida, Mexico. *Am J Trop Med Hyg.* 2011;84(3):489–496. 10.4269/ajtmh.2011.10-0654 21363990 PMC3042828

[15] Olano VA Matiz MI Lenhart A : Schools as potential risk sites for vector-borne disease transmission: Mosquito vectors in rural schools in two municipalities in Colombia. *J Am Mosq Control Assoc.* 2015;31(3):212–222. 10.2987/moco-31-03-212-222.1 26375902

[16] Echaubard P Thy C Sokha S : Fostering social innovation and building adaptive capacity for dengue control in Cambodia: a case study. *Infect Dis Poverty.* 2020;9(1):126. 10.1186/s40249-020-00734-y 32883345 PMC7469325

[17] Avila Montes GA Martínez M Sherman C : [Evaluation of an educational module on dengue and *Aedes aegypti* for schoolchildren in Honduras]. *Rev Panam Salud Publica.* 2004;16(2):84–94. 10.1590/s1020-49892004000800003 15357933

[18] Díaz-González EE Danis-Lozano R Peñaloza G : Schools as centers for health educational initiatives, health behavior research and risk behavior for dengue infection in school children and community members: a systematic review. *Health Educ Res.* 2020;35(5):376–395. 10.1093/her/cyaa019 32951047

[19] Overgaard HJ Alexander N Matiz MI : A Cluster-Randomized Controlled Trial to Reduce Diarrheal Disease and Dengue Entomological Risk Factors in Rural Primary Schools in Colombia. *PLoS Negl Trop Dis.* 2016;10(11):e0005106. 10.1371/journal.pntd.0005106 27820821 PMC5098800

[20] Jaramillo JF Vargas S Sarmiento-Senior D : [Sustainability of interventions to prevent dengue and diarrhea in rural schools in two municipalities in Colombia: a two-year post-project evaluation]. *Cad Saude Publica.* 2018;34(10):e00189017. 10.1590/0102-311X00189017 30365746

[21] Winch PJ Leontsini E Rigau-Pérez JG : Community-based dengue prevention programs in Puerto Rico: impact on knowledge, behavior, and residential mosquito infestation. *Am J Trop Med Hyg.* 2002;67(4):363–370. 10.4269/ajtmh.2002.67.363 12452490

[22] Chau T Fortin J Khun S : Practice what is preached? Dengue health education in Muan District, Khon Kaen Province, Thailand: Primary school children’s knowledge and reported practice. Australian Centre for International & Tropical Health and Nutrition. Brisbane: The University of Queensland. In: 2000.

[23] Khun S Manderson L : Community and school-based health education for dengue control in rural Cambodia: a process evaluation. *PLoS Negl Trop Dis.* 2007;1(3):e143. 10.1371/journal.pntd.0000143 18160981 PMC2154392

[24] Lennon J : Perceived self-efficacy to plan and execute an environmental action plan for dengue control among Filipino University students. *Dengue Bulletin.* 2007;31:160–165. Reference Source

[25] Parks W Lloyd L : Planning social mobilization and communication for dengue fever prevention and control: A step-by-step guide.In: World Health Organization, WHO/CDS/WMC/2004.2; 2004. Reference Source

[26] Andersson N Nava-Aguilera E Arostegui J : Evidence based community mobilization for dengue prevention in Nicaragua and Mexico ( *Camino Verde*, the Green Way): cluster randomized controlled trial. *BMJ.* 2015;351:h3267. 10.1136/bmj.h3267 26156323 PMC4495677

[27] Vector Borne Disease Control Programme: National Strategic Plan for Dengue Prevention and Control, 2016 - 2020.In: Ministry of Health and Sports, The Union of the Republic of Myanmar.; 2016.

[28] Outbreak News Today: Dengue in Myanmar: 23K cases in first 11 months of 2019.In: 2019; (accessed 10 Sep 2020). Reference Source

[29] Frontier Myanmar: The dreaded dengue on the rise.News article 27/09/2016. 2016; Accessed 15 Sep 2020. Reference Source

[30] Dengue infection prevalent in adults.

[31] Díaz-Menéndez M Esteban ET Ujiie M : Travel-associated chikungunya acquired in Myanmar in 2019. *Euro Surveill.* 2020;25(1):1900721. 10.2807/1560-7917.ES.2020.25.1.1900721 31937394 PMC6961262

[32] Ministry of Education: Education System in Myanmar (Brief Description of Primary, Secondary and Tertiary Education).In: Yangon, Myanmar: ASEAN Federation of Engineering Organisations (AFEO);2018. Reference Source

[33] Lin Z Tun Lin W Oo T : Community-based dengue source reduction interventions in two townships of Yangon region that significantly reduced entomological indices. *Dengue Bulletin.* 2012;36:206.

[34] Oo PM Wai KT Harries AD : The burden of dengue, source reduction measures, and serotype patterns in Myanmar, 2011 to 2015-R2. *Trop Med Health.* 2017;45:35. 10.1186/s41182-017-0074-5 29118655 PMC5667489

[35] Khin MM Saw L Soe A : Epidemiology of Dengue Haemorrhagic Fever in Myanmar, 1991-1998.Dengue Bulletin – WHO Regional Office for South-East Asia, New Delhi,1998;22. Reference Source

[36] WHO: Handbook for integrated vector management.In: WHO/HTM/NTD/VEM/2012.3. Geneva, World Health Organization,2012;12. Reference Source

[37] WHO: Global vector control response 2017-2030.In: Geneva: World Health Organization;2017. Reference Source

[38] Overgaard HJ Dada N Lenhart A : Integrated disease management: arboviral infections and waterborne diarrhoea. *Bull World Health Organ.* 2021;99(8):583–592. 10.2471/BLT.20.269985 34354313 PMC8319858

[39] WHO: Global Strategic Framework for Integrated Vector Management.In: Geneva: World Health Organization;2004. Reference Source

[40] Achee NL Gould F Perkins TA : A critical assessment of vector control for dengue prevention. *PLoS Negl Trop Dis.* 2015;9(5):e0003655. 10.1371/journal.pntd.0003655 25951103 PMC4423954

[41] Eiras AE Buhagiar TS Ritchie SA : Development of the gravid *Aedes* trap for the capture of adult female container-exploiting mosquitoes (Diptera: Culicidae). *J Med Entomol.* 2014;51(1):200–209. 10.1603/me13104 24605470

[42] Gruenewald DA : The Best of Both Worlds: A Critical Pedagogy of Place. *Educ Res.* 2003;32(4):3–12. 10.3102/0013189X032004003

[43] Wanich W : Place-Based Education in the United States and Thailand: With Implications for Mathematics Education.Appalachian Collaborative Center for Learning, Assessment, and Instruction in Mathematics Working Paper No. 33.2006. Reference Source

[44] Binot A Duboz R Promburom P : A framework to promote collective action within the One Health community of practice: Using participatory modelling to enable interdisciplinary, cross-sectoral and multi-level integration. *One Health.* 2015;1:44–48. 10.1016/j.onehlt.2015.09.001 28616464 PMC5462629

[45] Peerawaranun P Landier J Nosten FH : Intracluster correlation coefficients in the Greater Mekong Subregion for sample size calculations of cluster randomized malaria trials. *Malar J.* 2019;18(1):428. 10.1186/s12936-019-3062-x 31852499 PMC6921387

[46] WHO: Global Strategy for dengue prevention and control, 2012–2020.In: Geneva,2012. Reference Source

[47] Oo TT Storch V Madon MB : Factors influencing the seasonal abundance of Aedes (Stegomyia) aegypti and the control strategy of dengue and dengue haemorrhagic fever in Thanlyin Township, Yangon City, Myanmar. *Trop Biomed.* 2011;28(2):302–311. 22041749

[48] Chan AW Tetzlaff JM Gøtzsche PC : SPIRIT 2013 explanation and elaboration: guidance for protocols of clinical trials. *BMJ.* 2013;346:e7586. 10.1136/bmj.e7586 23303884 PMC3541470

[49] Knox TB Yen NT Nam VS : Critical evaluation of quantitative sampling methods for Aedes aegypti (Diptera: Culicidae) immatures in water storage containers in Vietnam. *J Med Entomol.* 2007;44(2):192–204. 17427686 10.1603/0022-2585(2007)44[192:ceoqsm]2.0.co;2

[50] Bangs MJ Focks DA : Abridged pupa identification key to the common container-breeding mosquitoes in urban Southeast Asia. *J Am Mosq Control Assoc.* 2006;22(3):565–572. 10.2987/8756-971X(2006)22[565:APIKTT]2.0.CO;2 17067066

[51] Warrilow D Northill JA Pyke A : Single rapid TaqMan fluorogenic probe based PCR assay that detects all four dengue serotypes. *J Med Virol.* 2002;66(4):524–528. 10.1002/jmv.2176 11857532

[52] Ito M Takasaki T Yamada KI : Development and evaluation of fluorogenic TaqMan reverse transcriptase PCR assays for detection of dengue virus types 1 to 4. *J Clin Microbiol.* 2004;42(12):5935–5937. 10.1128/JCM.42.12.5935-5937.2004 15583346 PMC535301

[53] Camerer C : Behavioral economics: reunifying psychology and economics. *Proc Natl Acad Sci U S A.* 1999;96(19):10575–10577. 10.1073/pnas.96.19.10575 10485865 PMC33745

[54] Mellor D Moore KA : The use of Likert scales with children. *J Pediatr Psychol.* 2014;39(3):369–379. 10.1093/jpepsy/jst079 24163438

[55] van Laerhoven H van der Zaag-Loonen HJ Derkx BH : A comparison of Likert scale and visual analogue scales as response options in children's questionnaires. *Acta Paediatr.* 2004;93(6):830–835. 10.1080/08035250410026572 15244235

[56] Wang C Burris MA : Photovoice: concept, methodology, and use for participatory needs assessment. *Health Educ Behav.* 1997;24(3):369–387. 10.1177/109019819702400309 9158980

[57] Shaffer R : Beyond the dispensary: African Medical and Research Foundation.Nairobi, Kenya.;1985. Reference Source

[58] Sahan K Pell C Smithuis F : Community engagement and the social context of targeted malaria treatment: a qualitative study in Kayin (Karen) State, Myanmar. *Malar J.* 2017;16(1):75. 10.1186/s12936-017-1718-y 28196536 PMC5310060

[59] Breitenstein SM Gross D Garvey CA : Implementation fidelity in community-based interventions. *Res Nurs Health.* 2010;33(2):164–173. 10.1002/nur.20373 20198637 PMC3409469

[60] Carroll C Patterson M Wood S : A conceptual framework for implementation fidelity. *Implement Sci.* 2007;2:40. 10.1186/1748-5908-2-40 18053122 PMC2213686

[61] Chard AN Freeman MC : Design, Intervention Fidelity, and Behavioral Outcomes of a School-Based Water, Sanitation, and Hygiene Cluster-Randomized Trial in Laos. *Int J Environ Res Public Health.* 2018;15(4):570. 10.3390/ijerph15040570 29565302 PMC5923612

[62] Zinszer K Caprara A Lima A : Sustainable, healthy cities: protocol of a mixed methods evaluation of a cluster randomized controlled trial for *Aedes* control in Brazil using a community mobilization approach. *Trials.* 2020;21(1):182. 10.1186/s13063-019-3714-8 32059693 PMC7023806

[63] European Commission: Guidelines on FAIR Data Management in Horizon 2020.In: Version 3.0, 26 July 2016: H2020 Programme, *European Commission, Directorate-General for Research & Innovation.* 2016. 10.25607/OBP-774

[64] Azevedo-Santos VM Vitule JR Garcia-Berthou E : Misguided strategy for mosquito control. *Science.* 2016;351(6274):675. 10.1126/science.351.6274.675 26912851

[65] Ministry of Health and Sports: National Guidelines for Dengue Prevention and Control, Ministry of Health and Sports, Myanmar.

[66] Campbell MK Piaggio G Elbourne DR : Consort 2010 statement: extension to cluster randomised trials. *BMJ.* 2012;345:e5661. 10.1136/bmj.e5661 22951546

[67] Hu Lt Bentler PM : Cutoff criteria for fit indexes in covariance structure analysis: Conventional criteria versus new alternatives. *Struct Equ Modeling.* 1999;6(1):1–55. 10.1080/10705519909540118

[68] Chen FF : Sensitivity of Goodness of Fit Indexes to Lack of Measurement Invariance. *Struct Equ Modeling.* 2007;14(3):464–504. 10.1080/10705510701301834

[69] Lincoln YS Guba EG : Naturalistic Inquiry.Newbury Park, CA: Sage Publications.;1985. Reference Source

[70] Talò C : Community-Based Determinants of Community Engagement: A Meta-Analysis Research. *Soc Indic Res.* 2018;140(2):571–596. 10.1007/s11205-017-1778-y

[71] Schairer CE Taitingfong R Akbari OS : A typology of community and stakeholder engagement based on documented examples in the field of novel vector control. *PLoS Negl Trop Dis.* 2019;13(11):e0007863. 10.1371/journal.pntd.0007863 31765377 PMC6901234

[72] Utarini A Indriani C Ahmad RA : Efficacy of Wolbachia-Infected Mosquito Deployments for the Control of Dengue. *N Engl J Med.* 2021;384(23):2177–2186. 10.1056/NEJMoa2030243 34107180 PMC8103655

[73] Johnson BJ Ritchie SA Fonseca DM : The State of the Art of Lethal Oviposition Trap-Based Mass Interventions for Arboviral Control. *Insects.* 2017;8(1)5. 10.3390/insects8010005 28075354 PMC5371933

[74] Mitchell-Foster K Ayala EB Breilh J : Integrating participatory community mobilization processes to improve dengue prevention: an eco-bio-social scaling up of local success in Machala, Ecuador. *Trans R Soc Trop Med Hyg.* 2015;109(2):126–133. 10.1093/trstmh/tru209 25604763 PMC4299531

