# Supplementary material for: School and community driven dengue vector control and monitoring in Myanmar: Study protocol for a cluster randomized controlled trial
Source: Wellcome Open Res. 2023 Dec 28;7:206. Originally published 2022 Aug 10. [Version 2] doi: 10.12688/wellcomeopenres.18027.2 (PMC10837613; doi:10.12688/wellcomeopenres.18027.2)
Supplement: Peer review history

# Reviewer report: Reviewer response for version 2

**DOI:** 10.21956/wellcomeopenres.22970.r71941

**Published:** 2024-02-02

**Authors:** Perran Ross

**Affiliations:** 1 The University of Melbourne, Melbourne, Victoria, Australia

The authors have addressed all my suggestions and I have no further comments.

Is the study design appropriate for the research question?

Yes

Is the rationale for, and objectives of, the study clearly described?

Yes

Are sufficient details of the methods provided to allow replication by others?

Partly

Are the datasets clearly presented in a useable and accessible format?

Not applicable

Reviewer Expertise:

Mosquito biology and control programmes

I confirm that I have read this submission and believe that I have an appropriate level of expertise to confirm that it is of an acceptable scientific standard.

# Reviewer report: Reviewer response for version 2

**DOI:** 10.21956/wellcomeopenres.22970.r71940

**Published:** 2024-01-02

**Authors:** I Made Dwi Mertha Adnyana

**Affiliations:** 1 Airlangga University, Surabaya, Indonesia

The main objective of this manuscript is to fill the limited literature related to efforts to reduce the entomological index and dengue cases through student and community involvement. Overall, the points of concern have been well addressed by the authors in accordance with previous comments. This protocol can be utilized as a pilot study for future researchers.

 The authors need to add a "limitation" section in the discussion section related to what are the limitations in the form of points and how this can spur further research in the future.".

Is the study design appropriate for the research question?

Yes

Is the rationale for, and objectives of, the study clearly described?

Yes

Are sufficient details of the methods provided to allow replication by others?

Yes

Are the datasets clearly presented in a useable and accessible format?

Yes

Reviewer Expertise:

Vector borne disease, eco-epidemiology, tropical and infectious disease, epidemiology, prevention and control of dengue

I confirm that I have read this submission and believe that I have an appropriate level of expertise to confirm that it is of an acceptable scientific standard.

# Reviewer report: Reviewer response for version 1

**DOI:** 10.21956/wellcomeopenres.19989.r63413

**Published:** 2023-11-28

**Authors:** Perran Ross

**Affiliations:** 1 The University of Melbourne, Melbourne, Victoria, Australia

With relatively few approaches to control the transmission of dengue virus by Aedes mosquitoes, there is a need to educate the public about the risks that mosquitoes pose to human health and ways for them to participate in suppressing their populations. There are a lack of studies investigating the impact of community engagement programmes on the incidence of dengue, and studies tend to focus on a single intervention rather than integrated approaches. Here, the authors have designed an intervention to equip school students with knowledge to contribute to the suppression of dengue in their own communities. Unfortunately, the current project appears to have been terminated by the donor, but the protocols are detailed and comprehensive and should be useful for others designing similar interventions in the future. I have one major concern about the use of guppies in the intervention (see below), but otherwise have only a few general suggestions for the authors to consider.

 There has been much success with engaging communities in conjunction with releases of Wolbachia-infected mosquitoes around the world. In some World Mosquito Program projects for instance, school students have been recruited to rear and release Wolbachia-carrying mosquitoes, where they are an active participant in dengue control programmes. Consider citing this an example of successful community engagement as part of a dengue control intervention (e.g. Costa et al. 2020 10.12688/gatesopenres.13153.2) ^1^.

 I am not convinced by the justification for the inclusion of larvivorous guppy fish in the study. Even if there is no risk of escape into the environment, I am not aware of much evidence for their effectiveness (see El-Sabaawi et al. 2016  10.1098/rsbl.2016.0590) ^2^. The authors state in the adverse events monitoring section that “guppies will be distributed to isolated water storage containers and are not likely to escape into the environment.” but this is in direct contrast to the methods within the intervention section, which states that students will be taught how to propagate the fish and that they will be provided for distribution to their households and communities. This would all but guarantee that they will escape into the environment. I suggest removing this component of the intervention.

 Methods – Cluster sizes are based on the size of the catchment, but is there any other justification for this? Is this an appropriate size and distribution given what we know about the dispersal ability and life cycle of dengue mosquitoes?

 Methods – Aside from source reduction, will students be educated about ways to avoid day-biting mosquitoes such as repellents and covering exposed skin?

 Interventions – The interventions are broad in scope but limited in detail. Will the authors attempt to ensure that the interventions are applied consistently across the treatment sites? For the vector control interventions the authors only list the four types of intervention and provide no additional information about the quantity and distribution of the traps and source reduction deployments.

Is the study design appropriate for the research question?

Yes

Is the rationale for, and objectives of, the study clearly described?

Yes

Are sufficient details of the methods provided to allow replication by others?

Partly

Are the datasets clearly presented in a useable and accessible format?

Not applicable

Reviewer Expertise:

Mosquito biology and control programmes

I confirm that I have read this submission and believe that I have an appropriate level of expertise to confirm that it is of an acceptable scientific standard, however I have significant reservations, as outlined above.

## References

[1] : How to engage communities on a large scale? Lessons from World Mosquito Program in Rio de Janeiro, Brazil. *Gates Open Res* .2020;4: 10.12688/gatesopenres.13153.2 109 10.12688/gatesopenres.13153.2 33103066 PMC7569240

[2] : Biodiversity and ecosystem risks arising from using guppies to control mosquitoes. *Biol Lett* .2016;12(10) : 10.1098/rsbl.2016.0590 10.1098/rsbl.2016.0590 28120806 PMC5095194

# Response

**Published:** 2023-12-19

**Authors:** Hans Overgaard

**Affiliations:** Norwegian University of Life Science, Norway

**Response to Reviewers for:** School and community driven dengue vector control and monitoring in Myanmar: Study protocol for a cluster randomized controlled trial   16 December 2023 **Reviewer 2** With relatively few approaches to control the transmission of dengue virus by Aedes mosquitoes, there is a need to educate the public about the risks that mosquitoes pose to human health and ways for them to participate in suppressing their populations. There are a lack of studies investigating the impact of community engagement programmes on the incidence of dengue, and studies tend to focus on a single intervention rather than integrated approaches. Here, the authors have designed an intervention to equip school students with knowledge to contribute to the suppression of dengue in their own communities. Unfortunately, the current project appears to have been terminated by the donor, but the protocols are detailed and comprehensive and should be useful for others designing similar interventions in the future. I have one major concern about the use of guppies in the intervention (see below), but otherwise have only a few general suggestions for the authors to consider.   There has been much success with engaging communities in conjunction with releases of Wolbachia-infected mosquitoes around the world. In some World Mosquito Program projects for instance, school students have been recruited to rear and release Wolbachia-carrying mosquitoes, where they are an active participant in dengue control programmes. Consider citing this an example of successful community engagement as part of a dengue control intervention (e.g. Costa et al. 2020 10.12688/gatesopenres.13153.2)1. Thanks for the suggestions. Community engagement is an important integral part of our intervention with specifically defined outcomes for students, parents and teachers (Table 2). We agree that Wolbachia is a suitable dengue intervention and recent results from trials show significant reductions in dengue cases in Indonesia (Utarini et al. 2021) and Colombia (Velez et al. 2023). However, a Wolbachia intervention requires are lot of preparatory work, for example mosquito producing facilities and community sensitization, which were not in place at the time our study was conceived. Furthermore, these trials, as well as the study of Costa et al., had not been completed when our study was developed. The purpose of our protocol was to propose a suitable, relatively simple and easily implemented integrated multi-method intervention in these settings. Implementers of future interventions might want to choose interventions that are more suited for their specific settings under future technological and knowledge advancements. Utarini A, Indriani C, Ahmad RA, Tantowijoyo W, Arguni E, Ansari MR, Supriyati E, Wardana DS, Meitika Y, Ernesia I, Nurhayati I, Prabowo E, Andari B, Green BR, Hodgson L, Cutcher Z, Rancès E, Ryan PA, O'Neill SL, Dufault SM, Tanamas SK, Jewell NP, Anders KL, Simmons CP; AWED Study Group. Efficacy of Wolbachia-Infected Mosquito Deployments for the Control of Dengue. N Engl J Med. 2021 Jun 10;384(23):2177-2186. Velez ID, Tanamas SK, Arbelaez MP, Kutcher SC, Duque SL, Uribe A, Zuluaga L, Martínez L, Patiño AC, Barajas J, Muñoz E, Mejia Torres MC, Uribe S, Porras S, Almanza R, Pulido H, O'Neill SL, Santacruz-Sanmartin E, Gonzalez S, Ryan PA, Denton JA, Jewell NP, Dufault SM, Simmons CP, Anders KL. Reduced dengue incidence following city-wide wMel Wolbachia mosquito releases throughout three Colombian cities: Interrupted time series analysis and a prospective case-control study. PLoS Negl Trop Dis. 2023 Nov 30;17(11):e0011713.   I am not convinced by the justification for the inclusion of larvivorous guppy fish in the study. Even if there is no risk of escape into the environment, I am not aware of much evidence for their effectiveness (see El-Sabaawi et al. 2016  10.1098/rsbl.2016.0590)2. The authors state in the adverse events monitoring section that “guppies will be distributed to isolated water storage containers and are not likely to escape into the environment.” but this is in direct contrast to the methods within the intervention section, which states that students will be taught how to propagate the fish and that they will be provided for distribution to their households and communities. This would all but guarantee that they will escape into the environment. I suggest removing this component of the intervention. Thanks for this suggestion. There is recent evidence of guppy fish effectiveness against mosquito larvae in containers from studies in Cambodia. We are aware of objections against the use of guppy fish for mosquito control due to potential environmental impacts. However, the evidence brought forward by El-Sabaawi et al. (2016) and other authors do not provide unambiguous conclusions. Therefore, we have initiated a systematic review to answer the question whether anthropogenic introductions of guppy fish impact faunal species diversity and abundance in natural aquatic habitats (Sasanami et al 2021). The review is ongoing. We have added the following sentence in the Discussion: “The justification of using guppy fish comes from recent evidence of from trials in Cambodia. Both immature entomological indices and population abundance of adult female Aedes mosquitoes were reduced in two community-driven integrated vector control interventions which included guppy fish, gravid-ovitraps, solid waste management, community education and engagement, and pyriproxyfen ^78, 79^. We believe that environmental risks, e.g., reduced biodiversity, of using guppy fish can be reduced by proper education on how to apply fish in containers in students’ homes. There is also an interesting educational component for students to understand biological concepts through fish breeding and development in school breeding aquaria.”   Bigio J, Braack L, Chea T, Set S, Suon S, Echaubard P, Hustedt J, Debackere M, Ramirez B, Prasetyo DB, Bunleng S, Wharton-Smith A, Hii J. Entomological outcomes of cluster-randomised, community-driven dengue vector-suppression interventions in Kampong Cham province, Cambodia. PLoS Negl Trop Dis. 2022 Jan 25;16(1):e0010028. doi: 10.1371/journal.pntd.0010028. Hustedt JC, Doum D, Keo V, Ly S, Sam B, Chan V, Alexander N, Bradley J, Liverani M, Prasetyo DB, Rachmat A, Shafique M, Lopes S, Rithea L, Hii J. Field Efficacy of Larvivorous Fish and Pyriproxyfen Combined with Community Engagement on Dengue Vectors in Cambodia: A Randomized Controlled Trial. Am J Trop Med Hyg. 2021 Sep 7;105(5):1265-1276. doi: 10.4269/ajtmh.20-1088. Sasanami, M., Hustedt, J., Alexander, N. et al. Does anthropogenic introduction of guppy fish ( *Poecilia reticulata*) impact faunal species diversity and abundance in natural aquatic habitats? A systematic review protocol. Environ Evid 10, 33 (2021).   Methods – Cluster sizes are based on the size of the catchment, but is there any other justification for this? Is this an appropriate size and distribution given what we know about the dispersal ability and life cycle of dengue mosquitoes? Our power calculations used township-level dengue incidence to establish a cluster size of 23 schools per study arm. The recruited schools in the townships include non-overlapping catchment areas, which will be carefully mapped. The size of catchment areas will be selected based on mosquito flight distance and other practical considerations. Optimally, there should be sufficient buffer zones between catchment areas to avoid contamination between intervention and control schools. To address this comment we have clarified the text in the “Study setting and participants” section as follows: “The delineation of school catchment area will consider mosquito flight distance from schools, but also providing sufficient buffer zones between adjacent catchment areas to avoid contamination between intervention and control schools.”   Methods – Aside from source reduction, will students be educated about ways to avoid day-biting mosquitoes such as repellents and covering exposed skin? Yes, this would be basic information that’s already included in the Aedes-free school program of Myanmar and would also be included in the educational part of the project. We have added “personal protection” in the education and knowledge transfer component in the Intervention section.   Interventions – The interventions are broad in scope but limited in detail. Will the authors attempt to ensure that the interventions are applied consistently across the treatment sites? For the vector control interventions the authors only list the four types of intervention and provide no additional information about the quantity and distribution of the traps and source reduction deployments. Yes, this is covered in the process evaluation where implementation fidelity and adaptation will assess how well interventions are implemented and if they were adapted by the users in any way (see Table 3). Details of the vector control interventions were not included in the original manuscript to reduce space and too much detail. We have a set of SOPs and descriptions of the vector control interventions and their deployment. We have added more detailed information in the vector control paragraph in the Interventions section.   Is the rationale for, and objectives of, the study clearly described? Yes Is the study design appropriate for the research question? Yes Are sufficient details of the methods provided to allow replication by others? Partly Are the datasets clearly presented in a useable and accessible format? Not applicable References 1. Costa GB, Smithyman R, O'Neill SL, et al.: How to engage communities on a large scale? Lessons from World Mosquito Program in Rio de Janeiro, Brazil. Gates Open Res. 2020; 4 109 PubMed Abstract | Publisher Full Text 2. El-Sabaawi RW, Frauendorf TC, Marques PS, et al.: Biodiversity and ecosystem risks arising from using guppies to control mosquitoes. Biol Lett. 2016; 12 (10):PubMed Abstract | Publisher Full Text   Competing Interests No competing interests were disclosed. Reviewer Expertise Mosquito biology and control programmes I confirm that I have read this submission and believe that I have an appropriate level of expertise to confirm that it is of an acceptable scientific standard, however I have significant reservations, as outlined above.

# Reviewer report: Reviewer response for version 1

**DOI:** 10.21956/wellcomeopenres.19989.r65114

**Published:** 2023-11-28

**Authors:** I Made Dwi Mertha Adnyana

**Affiliations:** 1 Airlangga University, Surabaya, Indonesia

This research aims to reduce the entomological index, including the incidence of dengue virus infection, by involving students in schools and the community. Overall this article is quite interesting, however there are several parts that need to be corrected to clarify the content of the article.

 Abstract

 "There are no effective vaccines or preventive drugs" this statement needs to be corrected, vaccines related to dengue have been developed and licensed (one of them is Dengvaxia® (CYD-TDV) how to respond to this?

 "We believe that students can be change agents for decentralised vector surveillance and sustainable disease control" What is your main conclusion from this protocol? and what implications can you recommend from this research later on, especially in areas with a high dengue burden?

 Introduction.

 "the incidence of reported dengue doubles about every decade" how big is this 2-fold increase? is the value constant or does it change? explicitly explained

 "One of the objectives of Myanmar's National Strategic Plan for Dengue Prevention and Control is to develop a comprehensive integrated training manual and guidelines, including community and school-based vector control"what programmes have been implemented by the local government so far? Whether there has been a significant change or no change at all, it is important to state this to support this sentence.

 "Simple single intervention approaches as practiced in the past have not been successful" what was the planned single intervention programme?

 The method.

 Is the researcher considering using focal fogging in the region? Do households and/or communities use insecticides and larvicides irregularly or even unwisely? this is one of the predictors that will interfere with this research, so it is important to consider.

 Sample size - "the number of adult mosquitoes in the area is expected to be large" this does need to be expressed in numbers to minimise ambiguity.

 Climate and environment - "Data on rainfall, temperature and humidity" in addition to these data, it is important to consider wind speed, barometric pressure, standardised precipitation index and sunshine duration, which also contribute to increased vector breeding in an area.

 "non-residential areas, vegetation and water bodies" what data is collected or recorded on these variables, especially water bodies such as rivers, ditches, drainage? Explain in detail.

 Discussion

 "Dengue transmission dynamics show strong spatial and temporal variation" what approach do you use? because in some areas this statement is not supported.

 "Although we cannot predict future outbreak patterns, we have selected townships with the highest number of cases in previous years and where no other dengue interventions have been implemented" temporal-spatial patterns, including identifying factors that play a very important role in events in the region.

Is the study design appropriate for the research question?

Yes

Is the rationale for, and objectives of, the study clearly described?

Yes

Are sufficient details of the methods provided to allow replication by others?

Yes

Are the datasets clearly presented in a useable and accessible format?

Yes

Reviewer Expertise:

Vector borne disease, eco-epidemiology, tropical and infectious disease, epidemiology, prevention and control of dengue

I confirm that I have read this submission and believe that I have an appropriate level of expertise to confirm that it is of an acceptable scientific standard, however I have significant reservations, as outlined above.

# Response

**Published:** 2023-12-19

**Authors:** Hans Overgaard

**Affiliations:** Norwegian University of Life Science, Norway

**Response to Reviewers for:** School and community driven dengue vector control and monitoring in Myanmar: Study protocol for a cluster randomized controlled trial   16 December 2023 **Reviewer 1** This research aims to reduce the entomological index, including the incidence of dengue virus infection, by involving students in schools and the community. Overall this article is quite interesting, however there are several parts that need to be corrected to clarify the content of the article. **Abstract** "There are no effective vaccines or preventive drugs" this statement needs to be corrected, vaccines related to dengue have been developed and licensed (one of them is Dengvaxia® (CYD-TDV) how to respond to this? Thank you, this text was written before the roll-out of the new dengue vaccines. We have amended the text to the following: “There are no preventive drugs and the newly introduced vaccines are not yet widely available. Control of dengue transmission still relies primarily on mosquito vector control.”   "We believe that students can be change agents for decentralised vector surveillance and sustainable disease control" What is your main conclusion from this protocol? and what implications can you recommend from this research later on, especially in areas with a high dengue burden? In the protocol we present a study design and rationale for the belief described above. With the research we hope to generate evidence of impact from engaging students as change agents. **Introduction.** "the incidence of reported dengue doubles about every decade" how big is this 2-fold increase? is the value constant or does it change? explicitly explained In the text we state “incidence of reported dengue doubles approximately every decade”, and cite a research study (reference 7) which presents this evidence. We emphasise that this is a rough approximation. Estimates of disease incidence rely on a range of factors which change over time including diagnostic criteria, access to health services and coverage and quality of surveillance systems. These vary greatly within and between countries and lead to wide uncertainties which are amplified at the global level. We have edited the text to explain this more explicitly: “, with estimates being highly uncertain due to a range of factors which change over time including diagnostic criteria, access to health services and coverage and quality of surveillance systems.” "One of the objectives of Myanmar's National Strategic Plan for Dengue Prevention and Control is to develop a comprehensive integrated training manual and guidelines, including community and school-based vector control" what programmes have been implemented by the local government so far? Whether there has been a significant change or no change at all, it is important to state this to support this sentence. Thank you. This sentence refers to the National Strategic Plan for 2016-2020 which was in place when this study was planned. We have edited the text to clarify this: “At the time of planning this study, one objective of the National Strategic Plan for Dengue Prevention and Control of Myanmar was to develop a comprehensive integrated training manual and guidelines” We do not have access to the updated National Strategic Plan, as it has not been made publicly available, and do not know what has been implemented by the local government since then. "Simple single intervention approaches as practiced in the past have not been successful" what was the planned single intervention programme?                 Thank you, we have added the following text: “, for example larval source reduction or fogging,” **Methods** Is the researcher considering using focal fogging in the region? Do households and/or communities use insecticides and larvicides irregularly or even unwisely? this is one of the predictors that will interfere with this research, so it is important to consider. Thank you, our intervention described in this protocol does not include focal fogging. Fogging is not an advisable intervention, since there is little evidence of a mosquito control effect. In addition, more insecticide use will inevitably lead to insecticide resistance. This is why our intervention does not include any insecticides. In response to the second question: the majority of insecticides for vector control used in households and communities are generally delivered by governmental vector control authorities and usually in response to an outbreak. Larvicides are probably less used on private initiatives.   Sample size - "the number of adult mosquitoes in the area is expected to be large" this does need to be expressed in numbers to minimise ambiguity. We do not have data on numbers of mosquitoes currently in the study area and these numbers were not used in the sample size calculation. We planned to collect this data (for the first time) at baseline and follow-up as outcome measures for the trial. Therefore we don’t need to express them in numbers, as suggested.   Climate and environment - "Data on rainfall, temperature and humidity" in addition to these data, it is important to consider wind speed, barometric pressure, standardised precipitation index and sunshine duration, which also contribute to increased vector breeding in an area. Thank you for the suggestion. We selected rainfall, temperature and humidity as they are easily measured, currently available from climate stations in Myanmar and have the strongest evidence for an association with dengue incidence. Wind speed, barometric pressure and sunshine duration are not routinely available and are not aware of evidence for a strong association with dengue incidence. Standardised Precipitation Index is not measured, it is calculated from rainfall data. "non-residential areas, vegetation and water bodies" what data is collected or recorded on these variables, especially water bodies such as rivers, ditches, drainage? Explain in detail. The full sentence in the Climate and environment section says: “Household densities (crowding) in school surroundings and school catchment areas will be collected from annotation of satellite images, as will land use including the presence of non-residential areas, vegetation, and water bodies.” Thus, the answer to the question is from satellite remote sensing images. Such data are freely available online. **Discussion** "Dengue transmission dynamics show strong spatial and temporal variation" what approach do you use? because in some areas this statement is not supported. Thank you for this comment. In most areas, including Myanmar, dengue transmission intensity varies greatly between locations and over time. We based this statement on published literature. There are many examples and we have added the following references to support this statement: Zaw W, Lin Z, Ko Ko J, Rotejanaprasert C, Pantanilla N, Ebener S, Maude RJ. Dengue in Myanmar: Spatiotemporal epidemiology, association with climate and short-term prediction. PLoS Negl Trop Dis. 2023 Jun 5;17(6):e0011331. doi: 10.1371/journal.pntd.0011331 Medina JRC, Takeuchi R, Mercado CEG, de Los Reyes CS, Cruz RV, Abrigo MDR, Hernandez PMR, Garcia FB Jr, Salanguit M, Gregorio ER Jr, Kawamura S, Hung KE, Kaneko M, Nonaka D, Maude RJ, Kobayashi J. Spatial and temporal distribution of reported dengue cases and hot spot identification in Quezon City, Philippines, 2010-2017. Trop Med Health. 2023 May 25;51(1):31. doi: 10.1186/s41182-023-00523-x. Phanitchat T, Zhao B, Haque U, Pientong C, Ekalaksananan T, Aromseree S, Thaewnongiew K, Fustec B, Bangs MJ, Alexander N, Overgaard HJ. Spatial and temporal patterns of dengue incidence in northeastern Thailand 2006-2016. BMC Infect Dis. 2019 Aug 23;19(1):743. doi: 10.1186/s12879-019-4379-3. PMID: 31443630; PMCID: PMC6708185.       "Although we cannot predict future outbreak patterns, we have selected townships with the highest number of cases in previous years and where no other dengue interventions have been implemented" temporal-spatial patterns, including identifying factors that play a very important role in events in the region. We don’t understand this comment. Is the rationale for, and objectives of, the study clearly described? Yes Is the study design appropriate for the research question? Yes Are sufficient details of the methods provided to allow replication by others? Yes Are the datasets clearly presented in a useable and accessible format? Yes Competing Interests No competing interests were disclosed. Reviewer Expertise Vector borne disease, eco-epidemiology, tropical and infectious disease, epidemiology, prevention and control of dengue I confirm that I have read this submission and believe that I have an appropriate level of expertise to confirm that it is of an acceptable scientific standard, however I have significant reservations, as outlined above.
